# Consolidated Overview of Notifiable Adverse Events in the U.S. President’s Emergency Plan for AIDS Relief’s Voluntary Medical Male Circumcision Program Through 2020

**DOI:** 10.1007/s11904-022-00636-8

**Published:** 2022-11-08

**Authors:** Todd Lucas, Caroline Cooney, Amber Prainito, Catherine Godfrey, Valerian Kiggundu, Anne Goldzier Thomas, Renee Ridzon, Carlos Toledo

**Affiliations:** 1 U.S. Centers for Disease Control and Prevention, Division of Global HIV and TB, HIV Prevention Branch, Atlanta, GA USA; 2 U.S. Department of State, Office of the U.S. Global AIDS Coordinator and Health Diplomacy, Washington, DC USA; 3 U.S. Agency for International Development, Global Health, Office of HIV/AIDS, Washington, DC USA; 4 U.S. Department of Defense HIV/AIDS Prevention Program, Defense Health Agency, San Diego, CA USA

**Keywords:** Male circumcision, Complication, Adverse event, HIV prevention

## Abstract

**Purpose of Review:**

Through December 2020, the U.S. President’s Emergency Plan for AIDS Relief (PEPFAR) supported more than 25 million voluntary medical male circumcisions (VMMC) as part of the combined HIV prevention strategy in 15 African countries. PEPFAR monitors defined adverse events (AEs) occurring within 30 days of VMMC through its notifiable adverse event reporting system (NAERS). All NAERS reports through December 2020 were reviewed to quantify AE type, severity, and relation to the VMMC procedure. Interventions to improve client safety based on NAERS findings are described.

**Recent Findings:**

Fourteen countries reported 446 clients with notifiable adverse events (NAEs); 394/446 (88%) were determined VMMC-related, representing approximately 18 NAE reports per million circumcisions. Fatalities comprised 56/446 (13%) with 24/56 (43%) of fatalities determined VMMC-related, representing 0.96 VMMC-related fatalities per million circumcisions. The remaining 390 NAEs were non-fatal with 370/390 (95%) VMMC-related. Multiple programmatic changes have been made based on NAERS data to improve client safety.

**Summary:**

Client safety is paramount in this surgical program designed for individual and population-level benefit. Surveillance of rare but severe complications following circumcision has identified pre-existing or new safety concerns and guided continuous programmatic improvement.

## Introduction

Male circumcision reduces a male’s risk of acquiring HIV through heterosexual transmission by approximately 60% [1–3]. Voluntary medical male circumcision (VMMC) programs are an important part of a comprehensive HIV prevention package for adolescent boys and men which also includes safer sex education, condom promotion, the offer of HIV testing services, linkage to HIV treatment for those testing positive, and the management of sexually transmitted infections [4]. VMMC has averted hundreds of thousands of new HIV infections in sub-Saharan Africa [5]. The U.S. President’s Emergency Plan for AIDS Relief (PEPFAR) has supported VMMC since 2007 and is the largest funder of global VMMCs. Through December 2020, PEPFAR has supported more than 25 million circumcisions in 15 priority countries, primarily in males aged 10 years and above, with a focus on 15–29-year-olds [6]. Recent modeling estimates that global VMMCs conducted through 2019 have averted 340,000 HIV infections in men and women and will avert about 1.8 million HIV infections by the end of 2030 [5].

VMMC is a preventive procedure conducted primarily in young healthy males. Although safety is paramount, there are inherent risks associated with VMMC because of its surgical nature. VMMC clients are screened for any health risks or contraindications to the procedure and receive pre-operative counseling on associated risks and benefits in order to provide informed consent. A review of VMMC trials in sub-Saharan Africa found a moderate and severe complication rate of about 2–4% under optimal clinical conditions [7]. Recently published VMMC-associated adverse event (AE) rates from well-established programs in multiple African countries ranged from 0.13 to 0.5% when passive reporting was utilized [8•, 9, 10••, 11, 12••]. When active AE surveillance is used, reported rates tend to be higher such as 3.1% in Zambia and 6.7% in Botswana [13, 14]. Underreporting is assumed during passive surveillance. This has been demonstrated in recent publications where active methods, such as chart reviews, antibiotic prescribing data, and prospective tandem clinical reviews where an auditing clinician examines postoperative clients alongside the VMMC provider, found higher AE rates. One study reported that site-level AE rates of 0.1–0.6% based on passive reporting increased to 1–8% when these same sites were assessed with tandem clinical reviews [10••]. Another found that a passively reported AE rate of 0.15% increased to 0.67% after record review, to 1.4% after additional antibiotic prescription review, and to 5.9% when prospective post-op visit observations were done [12••]. Although comparing AE rates in the literature is limited due to unclear severity criteria and heterogenous surveillance methods, infection and bleeding are consistently reported to be the most common AE types [9, 11, 12••, 13, 15•].

## Adverse Event Monitoring in VMMC programs

The recognition and proper management of AEs are critical components of any VMMC program. PEPFAR-supported VMMC programs follow standardized definitions and severity criteria for surgical and device-based circumcisions, as well as management of AEs, as detailed in the adverse event action guide for voluntary medical male circumcision by surgery or device 2nd edition [16]. PEPFAR monitors VMMC program quality and safety in several ways, including external quality assessments, PEPFAR Site Improvement Monitoring System visits, PEPFAR monitoring, evaluation, and reporting program data, continuous quality improvement activities, and monitoring of notifiable adverse events (NAEs) through an internal notifiable adverse event reporting system (NAERS). These are in addition to quality and safety monitoring and improvement activities that implementing partners (individual organizations funded to provide VMMC services) may have.

NAEs are a subset of severe AEs that occur within 30 days of the VMMC procedure, regardless of perceived relation to the procedure. These include (i) death, (ii) hospitalization for 3 or more days, (iii) injury to the glans or shaft of the penis, (iv) permanent or probable deformity or disability of the penis, (v) tetanus, and (vi) displacement of circumcision devices. Reports of NAEs meeting these criteria occurring after 30 days are also mandated if there is any evidence of relation to the VMMC.

Beginning in 2011, PEPFAR field teams were encouraged to report deaths and severe AEs on an ad hoc basis. The NAERS was created in 2014 focusing on reporting of deaths, and expanded in January of 2015, providing a systematic approach to reporting and analyzing NAEs within the PEFPAR program. PEPFAR-supported VMMC partners are required to report NAEs to PEPFAR for recordkeeping and analysis. All surveillance is passive, relying on reporting by organizations funded by PEPFAR to provide VMMC services using standardized forms and communication protocol with reporting from funded partners to PEPFAR field teams and headquarters. Reporting to PEPFAR of NAEs is in addition to in-country AE reporting requirements as determined by the Ministries of Health (MOH).

In-country VMMC staff investigate each NAE through clinical records and key informant interviews and discuss their findings with PEPFAR headquarters staff including possible contributing causes and potential mitigation plans as necessary. Based on these investigations, a determination is made of the relatedness of the NAE to the VMMC procedure on a scale of definitely related, likely related, likely unrelated, or definitely unrelated. A consultant physician makes the final determination, identifies areas for improvement, and provides recommendations for the prevention and management of potential future events. The NAERS is currently the sole system to centrally track and report NAEs across the PEPFAR VMMC portfolio. Starting in October 2021, the NAERS transitioned to reporting through PEPFAR’s data for accountability, transparency, and impact monitoring (DATIM) software system which will facilitate future analyses while preserving timely and secure NAE surveillance.

This article summarizes the NAEs that have been reported to PEPFAR from the first ad-hoc report in 2011 through December 2020 and discusses lessons learned and programmatic changes prompted by these lessons.

## Review Methods

PEPFAR maintains a central database of NAEs; this consisted of informal reporting on deaths and severe non-fatal AEs until 2014, added required death reporting in 2014, and then added the broader range of specified NAEs, along with a standardized reporting form, in 2015.^1^ This database records information such as client age, country, circumcision date, VMMC facility type, and provider characteristics. Each NAE report contains detailed clinical narratives which include information such as client medical history and examination results, procedural steps and operative findings, wound care practice, client complaints and postop exam findings, diagnostic workup, and adverse event management and outcome. Consulting physician impressions and recommendations are added to the final NAERS form for each reported case which is then disseminated back to in-country and U.S. government headquarters staff for review and implementation. Additionally, a high-level summary of program-wide NAERS reporting is shared with in-country PEPFAR staff on a quarterly basis.

All NAERS entries through December 2020, starting with the first ad-hoc report in 2011, were reviewed and described using total numbers and proportions. Data collection was funded by PEPFAR, and data analysis and interpretation were carried out by individuals supported by PEPFAR funding.

## Review Results

A total of 446 NAEs have been reported to PEPFAR through December 2020. From 2011 to 2014, before the full NAERS was established, approximately 7 million PEPFAR-supported VMMCs were performed and 19 NAEs were reported on an ad hoc basis [6]. The remaining 427 cases were reported from January 2015 through December 2020, after NAERS was fully established and over which time approximately 18 million PEPFAR-supported VMMCs were performed [6]. Of the 446 cases, 394 (88%) were determined to be related to the VMMC procedure, 37 (8%) were unrelated, and 15 (3%) were of unknown relationship to the VMMC procedure (Table 1). In general, the total volume of VMMCs increased year-over-year until 2020 when total volumes decreased, largely related to the effect of the COVID-19 pandemic [5].

**Table 1:** Notifiable adverse events reported by fatality status and relatedness to VMMC procedure (January 2011–Dec 2020)

Notifiable adverse events	Relatedness to VMMC procedure			
	*n* (% of total)	Related (% of row total)	Unrelated (% of row total)	Unknown (% of row total)
Total	446 (100)	394 (88)	37 (8)	15 (3)
Fatal	56 (13)	24 (43)	29 (52)	3 (5)
Non-fatal	390 (87)	370 (95)	8 (2)	12 (3)
Fatal notifiable adverse events	Relatedness to VMMC procedure			
	*n* (% of total)	Related (% of row total)	Unrelated (% of row total)	Unknown (% of row total)
Total	56 (100)	24 (43)	29 (52)	3 (5)
• Tetanus	13 (23)	13 (100)	0	0
• Infection	4 (7)	4 (100)	0	0
• Bleeding	4 (7)	2 (50)	2 (50)*	0
• Anesthesia toxicity	2 (4)	2 (100)	0	0
• Anaphylaxis	1 (2)	1 (100)	0	0
• Accidental and intentional injury†	7 (13)	0	7 (100)	0
• Infant cardiac/respiratory (e.g., congenital heart disease)	7 (13)	0	7 (100)	0
• Seizure disorder (status epilepticus)	3 (5)	0	3 (100)	0
• Malaria	4 (7)	0	4 (100)	0
• Acute GI pathology^‡^	3 (5)	0	3 (100)	0
• Undetermined^§^	5 (9)	1 (20)	1 (20)	3 (60)
• Other^¶^	3 (5)	1 (33)	2 (67)	0
Non-fatal notifiable adverse events	Relatedness to VMMC procedure			
	*n* (% of total)	Related (% of row total)	Unrelated (% of row total)	Unknown (% of row total)
Total	390 (100)	370 (95)	8 (2)	12 (3)
• Infection	171 (44)	170 (99)	0	1 (1)
• Bleeding	48 (12)	47 (98)	1 (2)	0
• Bleeding with secondary infection	18 (5)	18 (100)	0	0
• Fistula	51 (13)	51 (100)	0	0
• Glans injury (amputation or laceration)	40 (10)	36 (90)	0	4 (10)
• Tetanus	13 (3)	11 (85)	2 (15)	0
• Deformity not fistula or glans injury	24 (6)	23 (96)	0	1
• Device displacement	4 (1)	4 (100)	0	0
• Other^**^	16 (4)	10 (63)	5 (31)	1 (6)
• Unknown (undetermined/unrecorded)	5 (1)	0	0	5 (100)

^*^includes hemoptysis/thrombocytopenia (1) and leukemia (1)

^†^includes blunt head trauma (2), suicide (2), motor vehicle accident (1), accidental toxic ingestion (1), and drowning (1)

^‡^includes perforated duodenal ulcer (1), acute gastrointestinal bleed (1), and bowel obstruction (1)

^§^not enough information available to determine cause of death with relatedness based on clinical judgement of consulting physician if possible

^¶^includes hypertensive crisis (1), unspecified cerebral event (1), and brain abscess (1)

^**^examples include excessive skin removal, analgesic overdose, malaria, burns, and testicular torsion

Of clients with a reported NAE, 16/446 (4%) were among infants aged ≤ 2 months, 5/446 (1%) were among clients aged 2 months-9 years, 236/446 (53%) were among clients aged 10–14 years, 182/446 (41%) were among clients aged ≥ 15 years, and 7/446 (2%) were of unknown age.

### Fatal Adverse Events

Of the 446 total NAEs, 56 (13%) were fatal (Table 1). Of these 56 fatal NAEs, 24 (43%) were definitely or likely related to the procedure, 29 (52%) were definitely or likely unrelated, and the relatedness of death in 3 (5%) cases was unknown. With approximately 25 million PEPFAR-supported VMMCs performed through Dec 2020, this represents 0.96 VMMC-related fatalities reported per million circumcisions. Table 1 provides further details on the type of fatal NAE and its relatedness to the VMMC procedure. Thirteen of the VMMC-related deaths were due to tetanus; an approved elastic collar compression device was used in 4 of these 13 cases and standard surgical techniques were used in the other 9 cases. The other related deaths were due to infection (4), bleeding (2), anesthesia toxicity (2), anaphylactic shock (1), hypertensive crisis (1), and undetermined (1).

Causes of death for the 29 definitely or likely unrelated deaths were infant cardio-respiratory disease (e.g., congenital heart disease) (7), accidental or intentional injury unrelated to circumcision (7), malaria (4), seizure disorder (3), acute gastrointestinal pathology (3), and bleeding (2), and single cases of various causes which are further detailed in Table 1.

### Non-fatal Adverse Events

Of the 446 total NAEs, the majority (390, 87%) were non-fatal (Table 1). Of these 390 non-fatal NAEs, 370 (95%) were related to the VMMC procedure, 8 (2%) were unrelated to VMMC, and 12 (3%) were of unknown relatedness (Table 1). Further details on the 370 non-fatal NAEs related to VMMC are listed in Table 1. As expected with any surgical procedure, and consistent with reports in the VMMC AE literature, common NAEs include bleeding and infection. These bleeding and infection cases meet the “3 or more days of hospitalization” criterion and, therefore, represent very serious manifestations of these adverse event types.

Serious, non-fatal infection related to the VMMC procedure was reported in 171 cases. Many of these infections were described as necrotic, with some specifically diagnosed as Fournier’s Gangrene or necrotizing fasciitis by providers. Thirteen cases of non-fatal tetanus after VMMC were reported, of which 11 were deemed related to the VMMC. Of the 11 related non-fatal tetanus cases, two were via an elastic collar compression device while the remainder used a standard surgical method.

Extensive, but non-fatal, bleeding related to the VMMC procedure was seen in 66 clients, of which 18 had a secondary infection. In some cases, providers documented suspicion of a previously undiagnosed bleeding disorder; although local laboratory capacity for diagnostic testing was variable and it was not always possible to confirm. A few clients had a known bleeding disorder that was not disclosed at the time of initial screening for VMMC. Non-fatal injury to or deformity of the glans, shaft, or urethra, was reported in 115 cases, of which 51 were urethrocutaneous fistulas. Additional reported injuries included laceration of, or partial or complete amputation of, the glans (40 cases).

Finally, four device displacements were reported. A hard plastic collar-clamp circumcision device was pre-qualified by the World Health Organization (WHO) for male circumcision in 2015 and introduced across multiple countries during a series of implementation pilots and follow-on projects with enhanced safety monitoring. The device is designed to remain in place for 7 days prior to removal by a VMMC provider. Rare instances of the in-situ ring becoming detached early from the underlying tissue were reported during pilot projects. Due to the risk of significant bleeding, if the ring detaches, especially within the first few days after the procedure, this type of AE was added to the list of NAEs in 2020 so PEPFAR could more closely monitor device safety as use expands.

## Lessons Learned and Programmatic Improvements

NAERS has been a valuable asset in monitoring and evaluating the quality and safety of the PEPFAR VMMC program. Information gathered has contributed to changes and improvements in VMMC programs and has informed WHO guidance. Examples of these lessons learned are included in Table 2. With data combined from many countries, rare but serious NAEs that otherwise may be unnoticed can be detected and quantified. As part of the follow-up for each reported case, PEPFAR makes recommendations regarding program improvement (country-specific or across countries, as relevant) so that future NAEs can be reduced. Additional actions for improvement may also occur within specific countries through a MOH review of these same AEs or local changes at the implementing partner, facility, or individual provider levels.

**Table 2:** Lessons learned and programmatic improvements from the Notifiable Adverse Event Reporting System

NAE type	Lesson learned
Deaths	• There is a requirement for standardization of emergency resuscitation equipment at VMMC sites
Infection	• Even in the absence of known risk factors such as diabetes, there is potential for serious necrotizing infection in VMMC adverse events and a need for improved guidance on early diagnosis and management • Infections as the most common type of NAE contributed to PEPFAR’s increased emphasis on infection prevention and control guidance including additional resources on prevention and management of surgical site infections in VMMC programs
Tetanus	• In most VMMC priority countries, males over the age of 5 years may not have protective immunity to tetanus due to lack of booster doses. Several cases of tetanus were associated with application of traditional remedies to the surgical wound. Because traditional remedies may be contaminated with *Clostridium tetani* spores, all clients/parents need to be given written, graphic, and verbal instructions that they should not apply any substances to their wound during healing
	• Tetanus cases seen with elastic collar compression devices, in which the necrotic foreskin remained in situ for several days, triggered suspicion of a possible increased tetanus risk and led to the WHO risk analysis demonstrating a 30-fold increased risk with use of such devices when compared to conventional surgery and recommendation not to use such devices without documented appropriate vaccination
Bleeding	• Due to no prior medical/dental procedures in many young males, there is potential for the presence of unrecognized bleeding disorders to present at the time of VMMC and need for improved screening for bleeding risk
	• All episodes of post-operative bleeding should have close follow up and a second episode of post-operative bleeding should trigger referral to a healthcare setting with the capacity to provide blood transfusions, laboratory evaluation, and diagnosis of bleeding dyscrasias • Due to the familial X-linked nature of Hemophilia A, one of the most common bleeding abnormalities, providers should be advised not to perform VMMC on the brothers and cousins related through maternal aunts of clients with a bleeding disorder without proper preoperative evaluation
Penile injury	• Reports of glans injuries in males under 15 years signaled risk of forceps-guided technique; continued reports despite the requirement for use of dorsal slit method only signaled barriers to use of dorsal slit technique • Reports of fistulas, the vast majority of which occurred in clients under age 15, helped generate hypotheses on mechanisms of injury and interventions to minimize risk • Although still very rare, increased risk of penile injury in young adolescents prompted policy change to increase minimum age of eligibility to 15 years with mature genitalia

### Deaths

The early reports of deaths, whether determined related to the VMMC procedure or not, prompted standardization of an emergency resuscitation equipment list and the requirement for all facilities to have immediate access to these potentially life-saving medications and equipment. In-depth quality assurance tools were developed to assess compliance with this requirement along with adherence to multiple other quality standards.

### Infection and Tetanus

Infection is the most common NAE type reported in the system. Recognition of this has prompted increased attention to infection prevention and control activities in PEPFAR guidance and technical assistance, including the development of a surgical site infection prevention, diagnosis, and management guide for VMMC programs. Information gathered from NAERS, particularly on necrotizing wound infections led to the detailed discussion of the diagnosis and management of Fournier’s Gangrene in the adverse event action guide [16]. There is now emphasis on the need for early detection and aggressive treatment with both broad-spectrum antibiotics and emergent referral to a higher level of surgical care for debridement. Reported cases of infection following episodes of post-operative bleeding demonstrate the risk of infection posed by blood sequestered in tissue. Investigation and management of these cases led to guidance in the action guide recommending increased vigilance for signs and symptoms of infection in those with post-operative hematomas [16].

The reported cases of fatal and non-fatal tetanus following VMMC demonstrated the vulnerability of young males in countries that do not include tetanus booster doses to males in immunization schedules, including most of the 15 countries prioritized for VMMC [17, 18]. Reports of the use of traditional remedies on surgical wounds prior to the onset of tetanus led to enhanced counseling to avoid applying any substances to the wound and an emphasis on the clean care approach. Data shared with WHO allowed analysis demonstrating an increased risk of tetanus with the use of an elastic collar compression VMMC device compared to surgery. This led WHO to recommend the use of elastic collar compression devices only with assurance of protective immunity against tetanus through vaccination including administration of two doses, at least 4 weeks apart with the last dose at least 2 weeks prior to the procedure, in clients without known previous tetanus immunization [19]. Using data on risk, WHO and PEPFAR encouraged national immunization programs to add tetanus booster doses for males to their immunization schedules and encouraged the use of VMMC platforms for the administration of these boosters; PEPFAR also indicated support for MOH implementation of tetanus mitigation and treatment strategies for VMMC clients [19].

### Bleeding

Bleeding NAE cases highlight the importance of screening for potential bleeding disorders [20]. Because many clients may have never had a prior medical procedure, VMMC may be the first opportunity to recognize an undiagnosed bleeding dyscrasia. Following cases with severe bleeding likely due to a coagulopathy, and lack of recognition of such at the time of postoperative bleeding, screening materials were modified to include additional questions on individual and family histories of bleeding [21]. In these materials, providers were advised to re-question clients/parents upon presentation with any postoperative bleeding since a previously unrevealed bleeding disorder may be noted at the time of the bleeding. Due to the familial X-linked nature of Hemophilia A, one of the most common bleeding abnormalities, providers were advised not to perform VMMC on the brothers and cousins related through maternal aunts of clients with a bleeding disorder without proper preoperative evaluation.

In some cases, clients with significant bleeding were not referred for hospital admission until they presented several times with recurrent bleeding. Several clients demonstrated significant blood loss by the time of referral, demonstrating a need for more timely intervention. To ensure appropriate and timely referral of clients presenting with more than one episode of bleeding, algorithms for the management of postoperative bleeding were developed and included in the Action Guide [16]. Because clients with bleeding presented to VMMC clinics as well as clinics not staffed by providers trained in VMMC, separate algorithms with criteria for management and referral of clients with postoperative bleeding were developed for both VMMC clinics and other health care settings.

### Injuries

Initial observations of severe glans injuries and amputations in young clients following the use of the forceps-guided technique demonstrated the risk of injury with this procedure in young and sexually immature clients in whom there cannot be reliable palpation of the glans through the foreskin prior to foreskin excision. This led to WHO guidance and PEPFAR policy change to mandate the replacement of the widely used forceps-guided technique with the dorsal slit technique in clients under the age of 15 years. While this required widespread provider retraining, the seriousness of the glans injuries required this change to assure safety. When occasional cases continued to be reported with the incorrect use of the forceps-guided method, PEPFAR followed up with country teams and partners to ensure that providers were properly trained/re-trained in the use of dorsal slit surgical method and modified surgical instrument packs to include only the appropriate instruments for dorsal slit procedures while eliminating instruments necessary to perform the forceps-guided technique. Multiple glans injury cases submitted to NAERS occurred during early infant male circumcision (EIMC). The only method used, the Mogen clamp, was like the forceps-guided technique in that it did not allow glans visualization after clamping and before foreskin excision.

Analysis of fistula cases reported in NAERS also demonstrated an increased risk in young clients with immature genitalia [22]. Additional clinical information collected during NAERS reporting provided plausible hypotheses for mechanisms of urethral injury during circumcision, with age, likely as a proxy for physical maturity, as the most concerning. Although both glans injuries and fistulas were very rare occurrences, the increased risk in young clients led PEPFAR to change policy starting October 2020 to stop support for circumcising anyone under the age of 15 years, including EIMC, or a client of any age with immature genitalia^2^ [23, 24]. Prior to this decision, 10–14-year-old clients made up over 40% of PEPFAR’s annual VMMC volume.

### Limitations

While reporting into the NAERS is required, completeness or representativeness of the reporting is not known, and underreporting is suspected. Any estimates of program-wide NAE rates are limited by the unknown degree of underreporting and should be considered minimums. Thus, the NAERS is an important signal-generating process but is not exhaustive surveillance of all significant NAEs that occur in the context of PEPFAR VMMC programs. Differences in numbers between countries are likely due to differences in the number of VMMCs performed, and completeness of reporting, rather than overall differences in safety. Attempts to calculate NAE rates for comparison among programs based on these data could give misleading information and must acknowledge this limitation.

The increase in number of NAEs reported over time is likely due to increases in partner capacity to recognize and record NAEs, reporting compliance, and number of PEPFAR-supported VMMCs done each year. Efforts to increase reporting include reminders at regular meetings of PEPFAR country coordinators and at technical meetings, quarterly adverse event summaries shared with all PEPFAR teams, and assessment of reporting protocols during quality assurance assessments.

## Conclusion

Findings from the NAERS system have resulted in programmatic improvements from the individual site level to the multinational program level, including informing WHO guidelines and recommendations on VMMC safety. In-country providers acknowledge that the reporting is an added burden but note benefits of investigating cases as a means of triggering conversations assessing clinical management of cases, recognizing program problems, and implementing improvements.

As described in the recent WHO guidelines for preventing HIV through VMMC, male circumcision remains an efficacious HIV prevention option for adolescents aged 15 years and older and adult men [25]. VMMC programs should continue to be scaled up to meet the Joint United Nations Program on HIV/AIDS (UNAIDS) global goal of 90% male circumcision prevalence in older adolescents and men, thus, it is essential that VMMC program safety and quality be continuously assessed and improved [4]. Monitoring remains a key component to providing the safest VMMC program possible and PEPFAR continues to enhance the monitoring system through reporting reminders, and improved processes, forms, and analytic capabilities.

## Footnotes

1 Displacement of circumcision device (when a device intended to remain in place comes loose prior to the intended removal date) was added as an NAE in October 2020 if the AE severity was determined to be moderate or severe; the others have been designated NAEs since Jan 2015.

2 A small volume of device circumcisions in 13–14-year-old clients are supported by PEPFAR when accompanied by enhanced safety monitoring.
